# Unfavourable H‐CDR3 Loops in preB Cells Lead to Highly Expanded Plasma Cell Clones

**DOI:** 10.1002/eji.70200

**Published:** 2026-05-07

**Authors:** Alaitz Aranburu, Erik Engström, Erik Demitz‐Helin, Julia M. Scheffler, Alessandro Camponeschi, Timothy Sundell, Zhong Ni, Inga‐Lill Mårtensson

**Affiliations:** ^1^ Department of Rheumatology and Inflammation Research Institute of Medicine, Sahlgrenska Academy, University of Gothenburg Gothenburg Sweden; ^2^ Department of Chemistry and Molecular Biology University of Gothenburg Gothenburg Sweden; ^3^ Department of Clinical Immunology and Transfusion Medicine Sahlgrenska University Hospital Gothenburg Sweden; ^4^ School of Life Sciences Jiangsu University Zhenjiang China

**Keywords:** autoimmunity, B cell development, BCR, plasma cells

## Abstract

The immune system is essential for protection against invading pathogens. However, if it fails to distinguish between non‐self (pathogens) and self, autoimmunity can result. B cells recognise pathogens using their B‐cell antigen receptor (BCR), a membrane‐bound antibody. The variable region of the BCR contacts antigens via complementarity‐determining regions (CDRs). This region is encoded by immunoglobulin (Ig) V(D)J gene segments, which are randomly recombined during B‐cell development. Failure to counter‐select unfavourable H‐CDR3s is considered an underlying feature of autoimmunity. Here, we examined the effect of impaired pre‐BCR selection on plasma cells (PCs) using a model of autoimmunity characterised by elevated serum autoantibody levels. Our results demonstrate that the absence of a pre‐BCR leads to an increase in H‐CDR3s that are translated into unfavourable reading frames, encoding highly hydrophobic and/or basic amino acid residues, including a subset with extremely short H‐CDR3s. These features are not fully corrected by Ig light chains and persist in mature B cells. Ultimately resulting in the massive clonal expansion of PCs expressing a repertoire skewed towards extremely short H‐CDR3s that contain highly hydrophobic and/or positively charged residues. Consequently, preB cells with unfavourable H‐CDR3 features lead to the expansion of autoreactive PCs with the same features.

## Graphical abstract

In the absence of a pre‐BCR, a subset of mature B cells that expresses IgH chains with very short, hydrophobic and positively charged H‐CDR3 loops is generated. This results in massive clonal expansion and the development of autoreactive plasma cells exhibiting these characteristics.

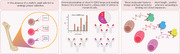

## 1 Introduction

A crucial feature of adaptive immunity is the ability of B cells to recognise and respond to invading pathogens with their membrane‐bound antibody, the B‐cell antigen receptor (BCR). T cell‐dependent responses lead to the activation of B cells, and the formation of germinal centre (GC) structures where B cells undergo clonal expansion, and their BCRs undergo somatic hypermutation (SHM) and class switch recombination (CSR) [1]. In the GCs, B cells are selected and differentiate into memory B cells (MBCs) or plasma cells (PCs). In autoimmune conditions, B cells are triggered by autoantigens but otherwise behave similarly to those in normal immune responses, although the selection process may differ [2]. In the mouse model of autoimmunity studied here, immunoglobulin heavy (IgH) chain selection is defective because preB cells are unable to express a precursor B‐cell receptor (pre‐BCR) due to the absence of the surrogate light chain (SLC^−/−^) [3, 4]. This also leads to defective B‐cell tolerance in the bone marrow (BM) and periphery, resulting in naive B cells with autoreactive properties [5]. Some of these cells are spontaneously activated, leading to the formation of T‐cell‐dependent GCs, MBCs and PCs, as well as elevated levels of serum autoantibodies [3, 6, 7, 8]. The latter are typical of those found in systemic lupus erythematosus (SLE), such as anti‐DNA and antinuclear antigen antibodies (ANA). Despite this aberrant selection process, no apparent disease develops.

A productively recombined VDJ encodes the variable region of the IgH chain, the region in close contact with the antigen, mainly through complementarity regions 1–3 (CDR1‐3). Unlike H‐CDR1 and H‐CDR2, which are encoded in the germline, the diversity of H‐CDR3 provides the basis for repertoire diversification. Our previous work has shown that, based on identical H‐CDR3s, GC B cells and MBCs in SLC^−/−^ mice are clonally related [9], reinforcing the idea of autoantigen‐specific MBCs and PCs. Moreover, the unusual features of H‐CDR3 in splenic PCs, including sequences with identical H‐CDR3s and the fact that some of them were strikingly short, were already apparent in our previous work [7]. Others have shown a distorted heavy chain repertoire in peripheral blood B cells, as well as in IgM‐expressing BM and splenic B cells in mice lacking the λ5 component of the pre‐BCR (λ5T mice), and have highlighted the presence of arginine, a positively charged (basic) amino acid (aa), in the central region of H‐CDR3 [10, 11]. The H‐CDR3 is often the target of study due to its topology and diversity, including its length and aa composition. These characteristics are largely determined by the D gene segment and the V‐D and D‐J joints during the early stages of B cell development [12].

Here, we studied antibody heavy chains expressed by PCs generated under autoimmune conditions and in the absence of active immunisation, to understand whether their H‐CDR3 characteristics are established early in B cell development.

## 2 Materials and Methods

### 2.1 Cell Sorting and High‐Throughput Sequencing of Ig Heavy Chains

The BM populations and spleen and BM PCs used in this study are the same as those used before [7]. Essentially, BM cells were collected and enriched for B cells using CD19 MACS beads (Miltenyi Biotec), followed by sorting of proB (B220^+^ c‐kit^+^), preB (B220^+^ c‐kit^−^ CD25^+^) and mature B cells (B220^+^ CD93^−^ IgM^+^) using six 8–10‐week‐old female WT or SLC^−/−^ mice. Spleen and BM PCs were isolated from a pool of five 7–8‐month‐old SLC^−/−^ mice. Spleen PCs were first enriched for by sorting CD19^low/+^ CD93^hi^ cells, followed by purity sorting using the same markers. BM PCs were first enriched for by sorting B220^−^ CD138^hi^ cells, followed by purity sorting using the same markers. The cells were sorted using a FACSAria (BD Biosciences) or a Synergy cell sorter (Sony Biotechnology), achieving purities of over 90%. The procedure for sequencing the heavy chains has been described previously [7], and the primers used are listed in Table S1.

### 2.2 International Immunogenetics Information System

Immunoglobulin heavy chain sequences were submitted to IMGT/HighV‐QUEST. We followed IMGT's unique numbering system, and the H‐CDR3 loop was defined as the sequence remaining after residues 104, 105 and 106, as well as 118 had been excluded [13]. All the properties, for example, length, charge and hydrophobicity, described here are referred to the H‐CDR3 loop.

### 2.3 Bioinformatics Analysis

PC sequences with 100% aa identity in the H‐CDR3 region were grouped into independent clones (Table 1). These were first stratified according to identical H‐CDR3s and separated into those with only one identical H‐CDR3 sequence (single) and those with >1 identical H‐CDR3 sequence (expanded). Within the ‘expanded’ category, those with a frequency of 1% or more of all PC sequences were termed ‘highly expanded’ (HE) PCs, while those with a frequency of <1% were identified but not analysed. H‐CDR3 landscapes, including the three categories (single, <1%, and ≥1%), were applied to all PCs from the SPL and BM, and visualised as treemaps using the ggplot2 (version 3.5.2) and treemapify (version 2.5.6) packages [14]. Each tile represents a unique H‐CDR3, with the tile's area proportional to its frequency (*n*).

**TABLE 1:** **Number of sequences**. Description of the number of productive and unique sequences from different cell types used in this study.

Organ and cell type	WT	SLC^−/−^	Figure
BM			
proB	2543	3929	Figure 1E,F; Figure 2A,B,D; Figure 4A,B (SLC^−/−^)
preB	6617	1063	Figure 1E,F,G; Figure 2A,B,D; Figure 4A,B,D (SLC^−/−^)
mB	12,105	418	Figure 1E,F,G; Figure 2A,B,D; Figure 4A,B,D (SLC^−/−^)
SPL PC			
H‐CDR3			
Single	—	424	Figure 1A,B,D; Figure 2A,B,E
Expanded <1%	—	1792	—
HE ≥1%	—	1083	Figure 1A,B,D,H; Figure 2A,B,E
H‐CDR3 + V_H_			
Single	—	827	Figure 4B
Expanded <1%	—	1652	—
HE ≥1%	—	820	Figure 1I; Figure 3A,C; Figure 4B
BM PC			
H‐CDR3			
Single	—	126	Figure 1A,B,D; Figure 2A,B,E
Expanded <1%	—	1238	—
HE ≥1%	—	2141	Figure 1A,B,D,H; Figure 2A,B,E
H‐CDR3 + V_H_			
Single	—	418	Figure 4B
Expanded <1%	—	1495	—
HE ≥1%	—	1592	Figure 1I; Figure 3A,C; Figure 4B

*Note*: The Figure column indicates the sequences used for the analysis in that particular figure and panel.

Abbreviations: BM, bone marrow; SPL, spleen; PC, plasma cell; HE, highly expanded; WT, wild type; SLC^−/−^, surrogate light chain deficient.

Subsequently, all PC sequences were stratified again based on identical H‐CDR3 and V_H_ usage (HE‐PC clones). Note that the number of single PC sequences increased in this analysis because some of these were assigned to the expanded category in the first stratification.

The physicochemical properties of the H‐CDR3 loop were calculated using the Peptides package in R [15]. The charge was calculated at pH = 7 using the Lehringer pKa scale [16]. The GRAVY hydrophobicity index was calculated using the Kyte–Doolittle scale [17]. The mean of each property was calculated for the different cell populations. Statistical comparisons were performed using the Mann–Whitney *U* test with Bonferroni correction for each population combination [18]. Hypermutated phylogenetic trees for selected PCs with shared H‐CDR3 and VH/JH rearrangements were constructed based on aligned VH regions using Dowser with the pml build option [19].

The FASGAI vectors of the H‐CDR3 loop were computed using the Peptides package in R [15, 20]. This set of six descriptors was chosen to reflect both structural features and hydrophobicity and electronic properties of the H‐CDR3 loop. The average value of each vector was calculated for each cell population and analysed using principal component analysis (PCA) to reduce dimensionality, as implemented in the R stats package.

### 2.4 Generation of Recombinant IgM Antibodies

The 7C9 and 7C9Mut antibodies, which are of the IgM isotype, were both generated by a commercial company (GenScript USA Inc). The recombinant 7C9 antibody has the same sequence as the previously characterised 7C9 hybridoma [6], and exhibits a germline rearrangement consisting of V_H_14‐2*01/D_H_3‐3*01/J_H_2*01 (H‐CDR3: CAR GRFDY W) and V_K_1‐110*01/J_K_5*01 (L‐CDR3: CSQSTHVPLTF). In contrast, the Mut7C9 antibody has not previously been characterised and carries an R‐to‐G mutation in the H‐CDR3 (H‐CDR3: CAR GGFDY W), exhibiting the same VDJ rearrangement as the 7C9 antibody.

### 2.5 HEp‐2 Stainings and Confocal Imaging

The samples were vortexed for 1 min and then centrifuged at 14,000×*g* for 10 min to remove potential precipitates. The HEp‐2 slide (Bio‐Rad, #26103) was allowed to reach room temperature before being placed in a humidity chamber. 25 µL of the undiluted, centrifuged recombinant antibody preparation was added to each well, and the slides were incubated for 30 min. All incubation steps were performed at room temperature in a humidity chamber and protected from light. After incubation, the slide was rinsed with PBS, followed by three 5 min washes with PBS. It was then carefully dried around the wells using the Wattmann paper provided. A dilution of 1:1000 of a goat anti‐human IgM secondary antibody in Alexa Fluor 488 (Invitrogen, #A‐21215) in PBS was added to each well (25 µL). The secondary antibody was then incubated for 30 min. After incubation, the slide was washed as described above. During the final wash, Hoechst (Invitrogen, H21486) was added at a final dilution of 1:10,000. After washing, the slides were dried and mounted using a homemade mounting medium based on Mowiol (Merck, #81381). Imaging was performed using a Leica SP8 confocal microscope with a 20× water objective and Leica software LASX software (vs 3.5.7). The z‐stack was obtained within a z‐range involving the cell layer (approximately 10–15 µm), with system‐optimised z‐steps. Maximum intensity projections were performed using LASX software. A digital zoom of 3.1× was used for the close‐ups. Orthogonal planes (xz and yz) were selected for each stack to visualise the nucleus of as many cells as possible. Images were converted to TIFF format using the LASX software. Black‐and‐white values were then adjusted in Adobe Photoshop (version 26.11), after which a scale bar and ROI were added.

## 3 Results

### 3.1 Short H‐CDR3 Loops Dominate the Expanded Plasma Cell Repertoire in the Absence of SL Chain

**FIGURE 1:**
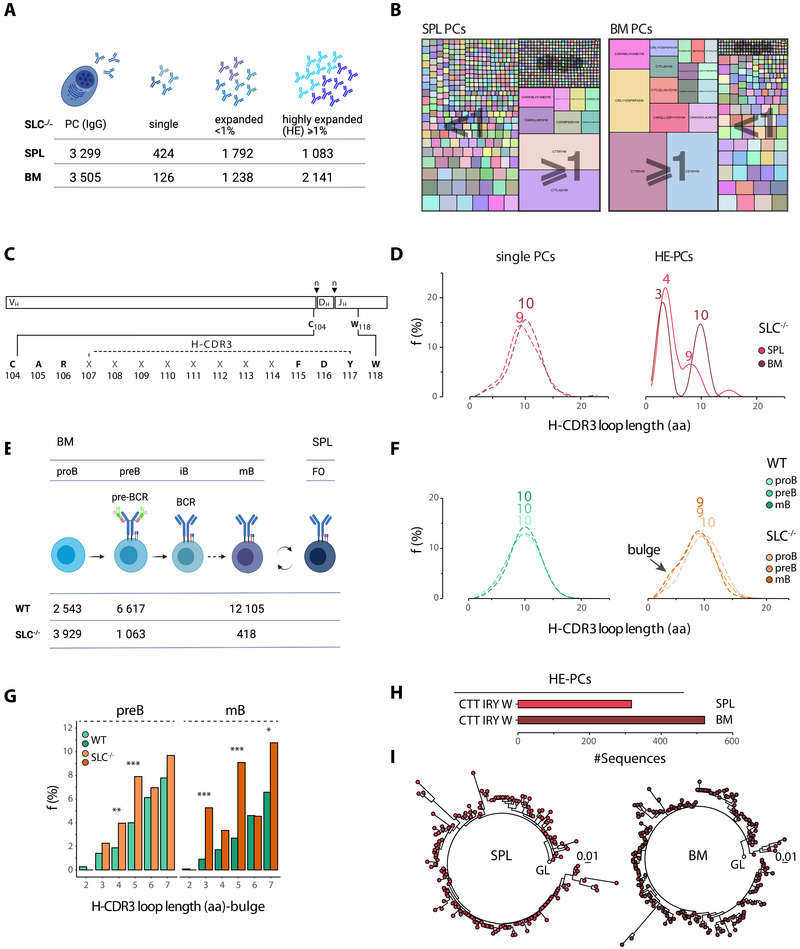
**SLC deficiency promotes the production of heavy chains with very short H‐CDR3 loops**. (A) Cartoon illustrating the classification of plasma cells (PCs) as single, expanded <1%, or highly expanded (HE) ≥1%, derived from the SPL or BM of SLC^−/−^ mice. (B) Treemaps showing the diversity and range of PCs classified as single, expanded <1% and highly expanded (≥1%) in the spleen (SPL) and bone marrow (BM) from SLC^−/−^ mice. Each tile represents a clonal VDJ rearrangement, with the size indicating the relative frequency of that particular clone. (C) Schematic representation of the VDJ recombination in the heavy (H) chain CDR3 and its variable loop (dashed line). IMGT numbering was used to mark the cysteine (C) and tryptophan (W) residues at positions 104 and 118, respectively, and N additions are indicated by n. The H‐CDR3 loop was defined by taking the IMGT junction sequence and excluding the three N‐terminal aa and the single C‐terminal aa from it, resulting in the central region. (D) Density histograms showing the frequency of H‐CDR3 loop lengths in single and highly expanded PCs from SPL (red) and BM (brown). (E) Cartoon showing B cell development in the BM and SPL: proB, progenitor B; preB, precursor B; iB, immature B; mB, mature B; FO, follicular B. (F) Density histograms showing the frequency of the H‐CDR3 loop length in BM populations. (G) Bar plot showing the distribution of short H‐CDR3 loops (2‐7 aa) in preB and mB cells, encompassing the so‐called bulge indicated in (F), from WT and SLC^−/−^ mice. (H) Number of sequences in one of the HE‐PC clones in the spleen and BM of SLC^−/−^ mice. (I) Circular lineage tree showing the splenic and BM H‐CDR3 clone CTT IRY W, which uses a V_H_14‐4. The mutations were calculated across the *IGHV* gene. GL, germline.

To determine the effect of the absence of the SL chain and the inability to express a pre‐BCR on the Ig heavy chains (HC) expressed by PCs, we analysed high‐throughput sequences from splenic and BM PCs. The total number of IgG PC sequences obtained from splenic and BM PCs was comparable, with over 3000 sequences obtained from each (Figure 1A; Table 1). These sequences were categorised as follows: ‘single’, representing those with only one unique H‐CDR3 sequence and presumably representing PCs that had not responded to autoantigen(s); ‘highly expanded’ (HE), consisting of sequences with a frequency of 1% or more of the PC sequences, meaning that each highly expanded clone would consist of at least 30 sequences; and finally, those named as ‘expanded <1%’, representing sequences falling between the other two categories, which were not considered further, except for Figure 1B. The IgG PC clones, that is, PCs expressing an H‐CDR3 that was 100% identical at the amino acid (aa) level, exhibited landscapes representing the three aforementioned categories (Figure 1B). However, of all the sequences, 87% in the spleen and 96% in the BM, were from clonally expanded cells, as was visually evident. As the mice had not been actively immunised, we hypothesised that these clones had likely expanded in response to autoantigen(s), although exogenous antigens may also have played a role. Due to its strong influence on antigen binding [21, 22], we examined the molecular features associated with the H‐CDR3, particularly the H‐CDR3 loop (Figure 1C).

Analysis of the two PC compartments revealed similar H‐CDR3 loop lengths with means of 9 and 10 aa in single clones from the spleen and BM, respectively (Figure 1D). In contrast, the majority of HE‐PCs from the BM and spleen exhibited extremely short H‐CDR3 loops with mean lengths of 3 and 4 aa, respectively. This is consistent with our previous work showing that PC populations in SLC^−/−^ mice are under selection at the level of H‐CDR3 length [7]. The remainder were of 9 and 10 aa length, spleen and BM, respectively, and thus of the same length as the respective single clones.

To determine whether the use of short H‐CDR3 loops is established early in B‐cell development in SLC^−/−^ mice, we examined the length distribution in BM proB, preB and recirculating mature naïve B cells (Figure 1E,F). In WT mice, the mean H‐CDR3 loop length was 10 aa, consistent with previous work [12]. The length in proB cells from SLC^−/−^ mice was also 10 aa; however, it was one aa shorter in preB cells, a length that was maintained in mature B cells. Additionally, a slight ‘bulge’ around 3 aa was observed in preB cells and mature B cells, which was one of the dominant lengths observed in expanded PCs. We therefore conducted a further analysis of the range of very short H‐CDR3 loops, spanning 2–7 aa, and found that loops of 4 and 5 aa were statistically significant in preB cells, whereas those of 3, 5 and 7 were so in mB cells (Figure 1G).

The next step was to verify that the clones that we had identified as expanded were indeed clonally expanded PCs. We therefore selected a clone that was expanded in both the spleen and the BM. This clone (CTT IRY W) had a short (3 aa) H‐CDR3 loop with a central arginine residue, and identical V_H_ and J_H_ recombination (Figure 1H). Lineage trees showed that the clones in the spleen and the BM (Figure 1I) had evolved from the original germline sequence following iterative rounds of mutations, which indicates exposure to antigen, presumably autoantigen. Thus, clonal expansion has indeed occurred, resulting in PCs with H‐CDR3 loops that are unusually short—a characteristic established already in preB cells, and hence associated with an inability to express a pre‐BCR.

### 3.2 Basic and Hydrophobic H‐CDR3 Loops due to Biased Reading Frame Usage

**FIGURE 2:**
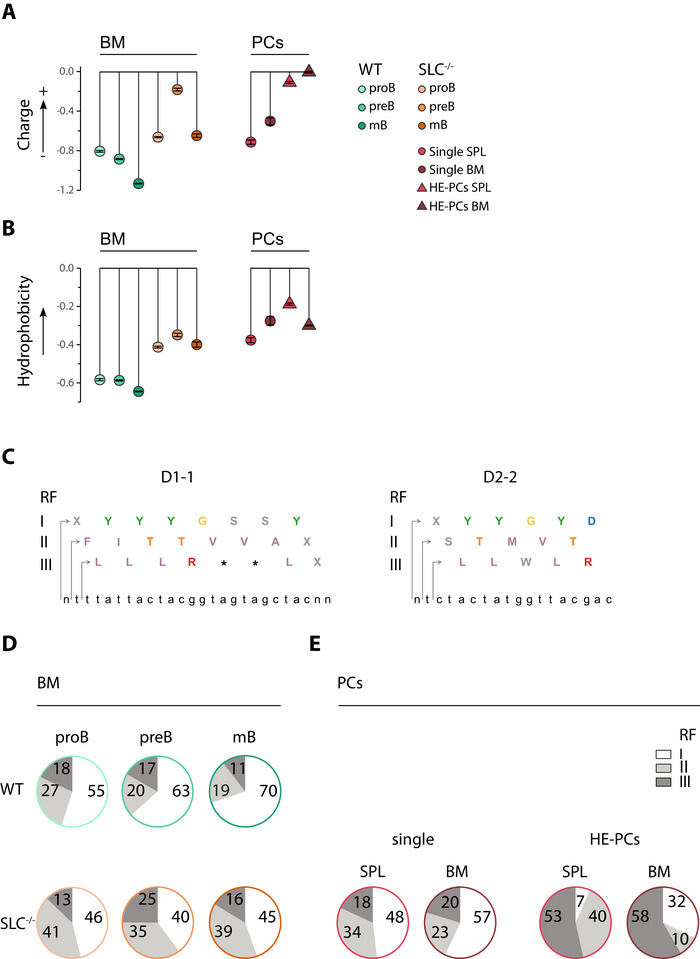
**Due to RF bias, the repertoire of pre‐BCR‐deficient preB cells consists mainly of basic and hydrophobic H‐CDR3 loops**. (A) Lollipop plots showing the mean net charge for BM populations and single and HE‐PCs from SLC^−/−^ mice. Values were calculated taking into account aa spanning the entire H‐CDR3 loop. WT vs. SLC^−/−^ proB padj = 3.74×10^−45^; preB padj = 0; mB padj = 2.23×10^−146^; SLC^−/−^ mB vs. spleen single PC padj = 4.27×10^−4^; SLC^−/−^ mB vs. BM single PC padj = 1. (B) Hydrophobicity was similarly calculated for the H‐CDR3 loop in the same populations. WT vs SLC^−/−^ proB padj = 2.31×10^−128^; preB padj = 1.99×10^−173^; mB padj = 4.42×10^−84^; SLC^−/−^ mB vs. spleen single PC padj = 1; SLC^−/−^ mB vs. BM single PC padj = 8.81×10^−5^. C) Example of the D1‐1 and D2‐2 gene segments in three possible reading frames (RFs) and their translation. Tyrosines (Y) are shown in green; hydrophobic residues such as alanine (A), leucine (L), valine (V), phenylalanine (F) and isoleucine (I) are shown in purple; arginine (R) is shown in red and threonine (T) is shown in orange. Asterisks represent stop codons. The germline nucleotide sequence is shown for each D gene segment. (D) Pie charts showing the distribution of the main RFs I (white), II (light grey) and III (dark grey) in BM B cells from WT and SLC^−/−^. (E) As in (D), but for single and HE‐PCs from SLC^−/−^ mice from SPL and BM. In (A) and (B), the Mann–Whitney *U* test with Bonferroni correction was used for statistical analysis.

We have previously shown that H‐CDR3s with a high number of basic aa (arginine and lysine) are more abundant in preB, mature B and GC B cells from SLC^−/−^ mice [3, 7]. To further investigate the properties of H‐CDR3s, we examined the net charge and hydropathy index found in the H‐CDR3 loops in the BM populations of WT and mutant mice. In SLC^−/−^ mice, preB cells in particular, but also mature B cells, showed a greater net positive charge compared with the corresponding WT populations (Figure 2A). Similar characteristics were also observed in the PC populations, regardless of the organ examined. Single clone values were comparable to those seen in mature B cells, while values in HE‐PCs were similar to those seen in preB cells from SLC^−/−^ mice. We then examined the hydropathy index, a score that measures the hydrophobicity of the aa, whereby a more positive calculated value indicates a more hydrophobic loop. The hydropathy index was increased in all BM populations of SLC^−/−^ mice compared with WT (Figure 2B). This feature was also evident in the HE‐PC populations of SLC^−/−^ mice, particularly in the spleen population, which showed highly hydrophobic H‐CDR3 loops. In conclusion, the BM populations of SLC^−/−^ mice have a higher net positive charge and hydropathy index in the H‐CDR3 loops than WT animals. These features are also present in HE‐PCs from SLC^−/−^ mice.

The nature of H‐CDR3 loops is partly determined by the reading frame (RF), into which a given D gene is translated to produce an in‐frame H‐CDR3 junction (Figure 2C). To study the three RFs, we followed the nomenclature established by Ichihara and Kurosawa [23]. A preference for RFI usage has been observed in mouse antibodies [23, 24], and it has been proposed that RFI is favoured by evolution to optimise the antigen‐binding repertoire, as it leads to the enrichment of tyrosine in H‐CDR3 (Figure 2C) [22, 25, 26]. The other two RFs are thought to be counter‐selected because they encode highly hydrophobic residues (valine in RFII, leucine in RFIII) and basic residues (arginine in RFIII) (Figure 2C). The distribution of reading frames in proB, preB and mature B cells in WT mice showed a decrease in RFs II and III, resulting in approximately 70% of mature B cells using RFI (Figure 2D), supported by previous studies [12]. A similar decrease in RFs II and III was observed at the same stages in SLC^−/−^ mice. However, due to their higher initial contribution, this resulted in a much lower proportion of mature B cells using RFI (45% vs. 70%).

We then examined RF usage in the PCs. Single clones showed a distribution most comparable to that of SLC^−/−^ mature B cells, particularly splenic PCs, of which fewer than half utilised RFI (Figure 2E). However, HE‐PCs showed a very different pattern. In splenic HE‐PCs, RFI was used by <10%, while RFII and RFIII were used by 40% and 53%, respectively. In BM HE‐PCs, RFI was used by 32%, while RFII and RFIII were used by 10% and 58%, respectively. Therefore, the RF usage observed in mature B cells from SLC^−/−^ mice is reflected in single PC clones, particularly those from the spleen. In contrast, RF usage in HE‐PCs is completely different, with a preference for RFII and/or III. This gives rise to H‐CDR3 loops containing basic and hydrophobic aa residues.

### 3.3 Most Expanded PCs Use Counter‐Selected RFs

**FIGURE 3:**
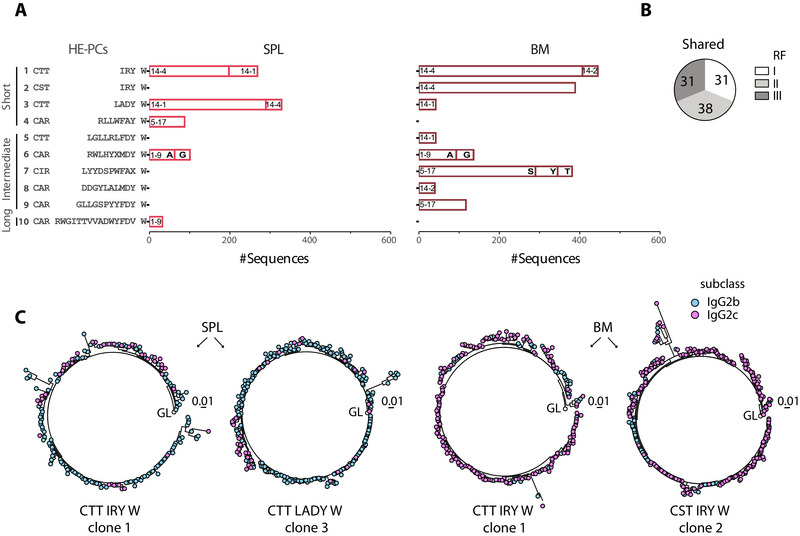
**The highly expanded PCs use counter‐selected RFs**. (A) The H‐CDR3 sequences of the HE‐PC clones were divided into a central loop, as defined in Figure 1B, and the aa remaining N‐ and C‐terminal to the loop. Further V_H_ annotation of the N‐terminal part of the H‐CDR3 loop helped identify smaller subclones within the main clones with identical H‐CDR3 aa sequences. Similarly, variations in the C‐terminal part of the H‐CDR3 loop were noted and considered as independent subclones. This gives a total of 8 and 12 in the spleen and BM, respectively. X = aa variation within the loop. (B) The RF distribution of the shared clones was calculated by considering only the RF of each shared clone. The definition of shared clone was based on the analysis shown in (A). (C) Circular lineage trees showing the isotype subclass distribution for the HE‐PC clones 1 and 3 in the SPL and in the BM. IgG2b (blue) and IgG2c (pink). The germline is indicated by a white circle. Clones 1 and 2 use V_H_14‐4, while clone 3 uses V_H_14‐1.

We then examined the HE‐PCs more closely. Initially, we considered only the H‐CDR3 loop, which showed that they could be divided into ten clones, that were found either in one or both compartments: four with short (<8 aa), five with intermediate (9–11 aa) and one with a long (15 aa) H‐CDR3 loop (Figure 3A; Table S2). On closer inspection, it became clear that some of these clones could be further divided according to V_H_ usage (HE‐PC clones). For instance, clone 1 could be subdivided into those with V_H_14‐4, which were highly expanded in both the spleen and BM (Figure 3A and 1G), and those with V_H_14‐1 and V_H_14‐2 in the spleen and BM, respectively (Figure 3A). Clone 2 contained the same loop as clone 1 but had a different 5’ end, CST instead of CTT. As this clone also used V_H_14‐4, and this V_H_ encodes CTT in the germline, it is likely to be the result of somatic hypermutation of CTT to CST. However, the J_H_ is different in clone 2, which makes it a distinct clone. Clone 3 mainly used V_H_14‐1 in both spleen and BM. Clones 5 and 8 also used members of the V_H_14 family. In fact, a significant proportion of sequences belonging to the expanded clones in both PC compartments expressed a member of the V_H_14, accounting for approximately half of these sequences, most of which had a short H‐CDR3 loop.

We identified two additional V_H_ genes among the HE‐PC clones, V_H_5–17 and V_H_1–9. V_H_5–17 accounted for almost a quarter of the HE‐PC clones in BM. Clone 7 with V_H_5–17 can be further subdivided into three clones differing by a replacement mutation of the germline Y to S or T. As the clone with S was the most expanded, this suggests that this mutation is beneficial. Clone 6 with V_H_1–9 represents two clones that differ by one aa, which is most likely a replacement mutation from the germline A to G. Clone 10, with V_H_1–9, is the only clone with a long loop and is the least expanded and only found in the spleen. Upon considering the H‐CDR3 loop alongside the V_H_ gene, the HE‐PCs could be viewed as 16 clones rather than 10. Analysis of RF usage in the 16 (shared) clones revealed that all three RFs were used in almost equal proportions (Figure 3B), resembling the ratio in SLC^−/−^ preB cells.

### 3.4 HE‐PC Clones With Short H‐CDR3 Loops Preferentially Express IgG2c in BM and IgG2b in Spleen

As the degree of expansion of the clones varied between the spleen and BM, we asked whether this was related to the IgG subclass. Overall, the dominant isotypes in both the spleen and the BM were IgG2b and IgG2c, with the IgG1 (2% spleen; 6% BM) and IgG3 (8% spleen; 5% BM) subclasses representing a minority. However, the distribution of the IgG2b and IgG2c subclasses appeared to be asymmetric, particularly in the HE‐ PC clones with short H‐CDR3 loops. This was evident in clone 1, which had a very short loop. Although it was highly expanded in both compartments, IgG2b was dominant in the spleen and IgG2c in the BM (Figure 3C). Two other clones with short loops followed a similar pattern: clone 2 was highly expanded in the BM and of the IgG2c isotype, while clone 3 was highly expanded in the spleen and of the IgG2b isotype. Therefore, differences in isotype distribution appear to be linked to the PC compartment rather than the degree of PC expansion.

### 3.5 The Incomplete Counter‐Selection of Molecular Features in the H‐CDR3 Loop Drives the Expansion of Plasma Cells

**FIGURE 4:**
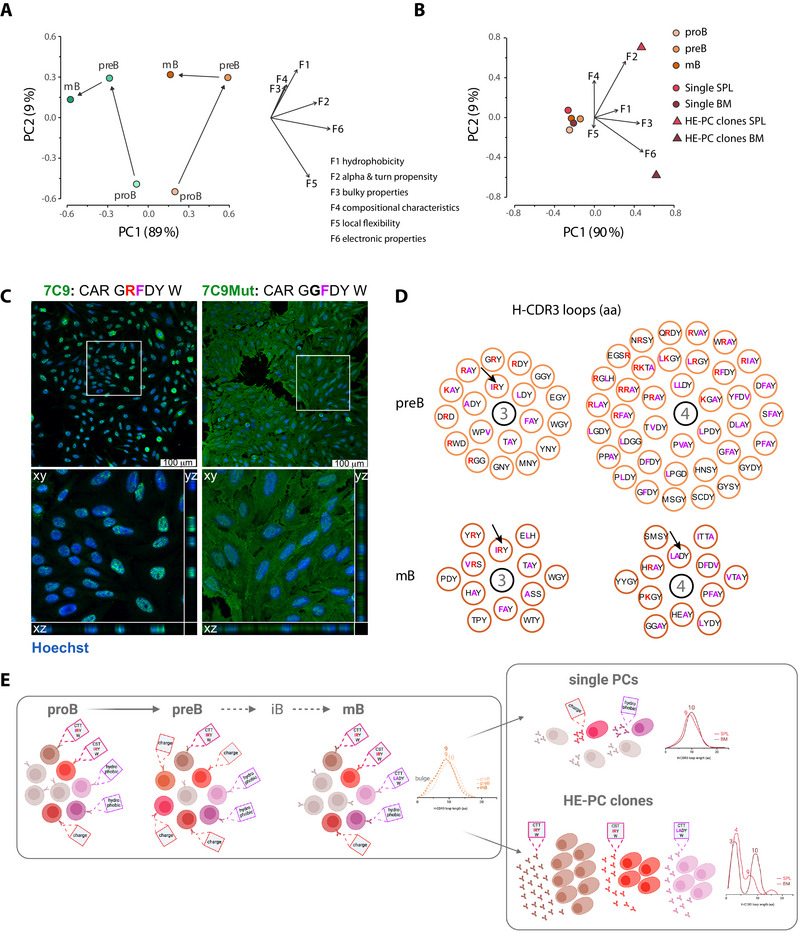
**The molecular features of the H‐CDR3 loop are altered in the preB, mB and PCs of mice lacking a pre‐BCR**. (A) PCA using H‐CDR3 loop properties for BM B cells from WT (green) and SLC^−/−^ (brown) mice. (B) PC clones, single and HE, were considered alongside BM B cells from SLC^−/−^ mice. (C) Confocal images were obtained by incubating HEp‐2 slides with either the 7C9 or the 7C9Mut recombinant antibodies, along with AF488‐coupled goat anti‐IgM and Hoechst. The overview images correspond to maximum‐intensity projections of merged staining. The cells within the marked square were digitally enlarged and analysed using a z‐stack to help discriminate between nuclear and cytoplasmic staining in the xz and yz planes. (D) The H‐CDR3 loops of lengths 3 and 4 aa are depicted in preB and mB cells of SLC^−/−^ mice. Arrows indicate the IRY and LADY H‐CDR3 loops. (E) Model that summarises the properties of the H‐CDR3 loops found in the BM and PC populations of SLC^−/−^ mice. ProB cells: hydrophobic (purple tag) and basic (red tag) loops are prominent. PreB cells: positive charge is more prominent, length is reduced, including a subset with very short loops. mB cells: hydrophobic and positive charge are similar to proB cells, length is similar to preB cells. Single PCs: hydrophobic and positive charge, and length, but not the subset with very short loops areops, are similar to mB. HE‐PC clones: hydrophobic and positive charge, very short length. Positively charged residues are shown in red and hydrophobic residues in purple.

Due to the significant differences observed in the H‐CDR3 repertoire during B cell development when comparing WT and mutant mice, as well as in the HE‐PCs in the latter, we used the FASGAI (Factor Analysis Scales of Generalized Aa Information) method to characterise the H‐CDR3 loops further. This method groups 335 physicochemical properties of the 20 natural aas into six factors (F1–F6): F1, hydrophobicity; F2, alpha and turn propensities; F3, bulky properties; F4, compositional characteristics; F5, local flexibility; and F6, electronic properties [27]. PCA analysis was performed to visualise and interpret the FASGAI results. In the BM, PC1 accounted for almost 90% of the variance in the data and exhibited a significant impact as cells progressed from proB to preB and ultimately to mature B cells in WT mice (Figure 4A). PC2 also had some influence, though it accounted for less than 10% of the variance in the data, particularly at the proB‐to‐preB transition. In SLC^−/−^ mice, proB cells were relatively similar to their WT counterparts in PC2, but rather different in PC1. As the cells progressed to preB cells, this difference became more pronounced, with the cells adopting a distant position from their WT counterparts. Despite an apparent effort to ‘correct’ for the deviation, mature B cells still exhibited significant PC1 displacement from their WT counterparts as they progressed from preB cells. The major molecular features that dictate these transitions are F6 and the closely associated F2, which relate to the formation of secondary structures. Higher values indicate a greater tendency towards alpha helix formation. F1 and F5 also play a role, albeit to a lesser extent (Figure 4A). This demonstrates that selection occurs at each transition, highlighting the significant role of the H‐CDR3 in these events, its reliance on the SL chain and the fact that it cannot be fully compensated for by bona fide Ig light chains.

Next, we investigated the H‐CDR3 properties of single and HE‐PC clones. Whereas the single PCs and BM populations, especially the mB, were close, the HE‐PC clones were separated from all of them, which was driven by F1, F2, F3, and F6 (Figure 4B). As was also observed in preB cells from pre‐BCR‐deficient mice, F6 and F2 predominantly facilitate the separation, followed by F1 and F3. These features, which are associated with hydrophobicity and bulky properties, are linked to aas such as arginine (positive charge), phenylalanine (hydrophobic), and tryptophan and tyrosine. These aa are present in the H‐CDR3 loops of HE‐PC clones, and are thus likely the major contributors to the separation observed between the populations (Table S2). The large number of sequences constituting the HE‐ PC clones could affect the property calculations since an identical loop is counted as many times as the size of the clone (Table 1; Table S2). We therefore reanalysed the data, this time considering only the original clones rather than all their members. However, the results were very similar, indicating that F1–F3 and F6 are the primary forces that separate the H‐CDR3 repertoire of the HE‐PC clones from that of the single clones and the BM populations in SLC^−/−^ mice (data not shown).

Overall, our results demonstrate that short hydrophobic and charged H‐CDR3 loops constitute a significant proportion of the PC repertoire in SLC^−/−^ mice. To investigate the potential of such loops to drive autoreactivity, we generated a recombinant antibody that replicates some of these features. To this end, we took advantage of a hybridoma, 7C9, with a short, positively charged H‐CDR3 loop (G**R**FDY), which we previously showed binds to nuclear antigens, most likely the Smith (Sm) antigen [6]. After sequencing also the Ig light chain, two recombinant antibodies were synthesised: one with the original sequence (7C9), and a mutated counterpart (7C9Mut) with the same short loop but neutral charge (G**G**FDY). The recombinant 7C9 reproduced the previously observed speckled ANA pattern (Figure 4C). In contrast, the mutant showed a very different pattern, namely a cytoplasmic pattern, losing the speckled nuclear pattern entirely.

To further emphasise that the observed expansion in PCs is likely due to features such as short hydrophobic and/or positively charged H‐CDR3 loops, we examined these characteristics specifically in preB and mB cells. We found that short loops did indeed contain an enrichment of hydrophobic and/or positively charged aa residues (Figure 4D). Moreover, we identified the IRY and LADY loops seen among the HE‐PC clones. These results support the idea that interaction with autoantigens recognised by these autoreactive features leads to excessive expansion of PCs.

We conclude that, while B‐cell development in SLC^−/−^ mice follows the normal pathway, their H‐CDR3 repertoire differs significantly from that of WT mice. This segregation is primarily driven by the presence of hydrophobic and basic aa residues in the H‐CDR3 loops. Together with the extremely short loops present in preB cells that are not fully amended in mB cells, these autoreactive properties cause the substantial expansion of PCs in the BM and spleen of SLC^−/−^ mice (Figure 4E).

## 4 Discussion

The antibody µHC undergoes selection during B cell development in the BM of adults, which significantly affects the nature and outcome of the BCR repertoire expressed by developing mature B cells. It also affects immune and autoimmune responses, impacting the repertoire expressed by PCs. As we demonstrate here, one determining factor in this selection process is the length of the H‐CDR3 loop. In SLC^−/−^ mice, the loop is reduced by one aa in preB and mB cells. An accumulation of a subset with very short loops was also observed in preB and mB cells of the mutant mice. In HE‐PCs, a subset with very short loops outnumbered those that were 9–10 aa. By contrast, those with short loops were barely present in single PCs, most of which were 9–10. The importance of the SL chain in selecting H‐CDR3 length is supported by observations showing that peripheral blood B cells with H‐CDR3 loops shorter than 8 aa residues are more prevalent in λ5T mice compared with WT mice [10].

The H‐CDR3 can be read in different RFs with a preference for RFI in WT mice [23, 24]. However, we observed an overrepresentation of RFII usage in SLC^−/−^ mice at all developmental stages. Further evidence supporting a lack of RFII suppression comes from λ5T mice [28, 29]. It was also observed that this selection was more prevalent in D2‐containing sequences, with increased D2 usage. A significant decrease in D1 (primarily D1‐1) usage and an increase in D2 usage are also evident in SLC^−/−^ mice (Figure S1). We also observed an overrepresentation of RFIII in preB cells, consistent with our previous findings [3]. Using the less favourable RFs impacts the antibody repertoire produced by PCs in SLC^−/−^ mice. There was a difference between single PCs and HE‐PCs: the former appeared to adopt RF usage similar to that of the main population of mB cells, while the latter favoured RFII and RFIII.

RFI leads to a high proportion of tyrosine residues. It has been proposed that the presence of tyrosine at specific positions in the H‐CDR3 loop enhances binding to VpreB1, resulting in a more stable pre‐BCR [30]. However, RFII and RFIII are overrepresented in preB and mB cells from SLC^−/−^ mice, resulting in a high proportion of highly hydrophobic (leucine/valine) and basic (arginine) aa residues, respectively. An overrepresentation of loops containing arginine has also been observed in IgM‐expressing BM cells from λ5T mice, as has the CTT IRY W loop [11]. Failure to enrich for tyrosine has knock‐on effects at subsequent stages, meaning that mB cells inherit these features and pass them on to subsequent PC populations. This is evident in the HE‐PC clones in SLC^−/−^ mice, where most contain an H‐CDR3 loop with either leucine and/or valine, and where over half contain a loop with an arginine. However, very few have tyrosine in the optimal position in the loop.

In conclusion, the absence of the SL chain leads to the early establishment of autoreactive features that are passed on to PCs in the form of short, hydrophobic and/or basic H‐CDR3 loops. This may explain the high levels of autoantibodies in this autoimmune model. This suggests that bona fide Ig light chains are unable to correct for unfavourable H‐CDR3s. Classically, longer H‐CDR3 lengths have been associated with poly/self‐reactivity [31, 32]. Interestingly, however, shorter H‐CDR3s have been reported among naïve B cells in humans with autoimmune diseases [33], as well as in PBMCs from patients with systemic sclerosis [34] and systemic lupus erythematosus [35]. Studies have shown that hypomorphic biallelic mutations in the *RAG1* gene can compromise VDJ recombination in patients with Omenn syndrome (OS). This results in a marked decrease in diversity and an uneven distribution of clonotypes, which are associated with clonotypic expansions. In this regard, our data on PCs, in which over 90% of sequences are assigned to a few clones, resemble those described in OS with clonal skewness, where a few clones are highly represented [36]. The same study also showed that the H‐CDR3 profile in OS is abnormal, with the complexity score, skewness and kurtosis being significantly different from those of control subjects. The study also observed a high representation of very short H‐CDR3s, which were enriched overall in both hydrophobic residues, such as leucine, and hydrophilic residues, such as the negatively charged aa aspartic acid.

## Author Contributions

I.‐L.M. and A.A. designed the experiments, A.A., E.E., and E.D.H. analysed the data, and I‐LM, A.A., and E.E. interpreted the results. J.M.S. performed the HEp‐2 stainings and acquired the confocal images. A.C., T.S., and Z.N. contributed with fruitful discussions. I.‐L.M. and A.A. wrote the manuscript.

## Funding

Funding for this project was kindly provided by Swedish Science Research Council, 2021‐01150, 2024–03753 (I.‐L.M.), King Gustav V Stiftelse, FAI‐2023‐1033, FAI‐2024‐1147 (I.‐L.M.), FAI‐2024‐1132 (A.A.); the Swedish Cancer Society, 22 2467Pj (I.‐L.M.); ALF agreement; the Swedish government and the county council, ALFGBG‐277797, ALFGBG‐1006883 (I.‐L.M.); IngaBritt and Arne Lundbergs Research Foundation LU2015‐093, LU2019‐0031 (I.‐L.M.); Patient Association for Rheumatic Diseases, R‐94129 (I.‐L.M.), R‐995343 (A.A.); Wilhelm and Martina Lundgrens Foundation 2023‐SA‐4322 (A.A.), 2024‐SA‐4632, 2025‐SA‐5038 (A.C.); Stiftelsen Mary von Sydows donationsfond, 2025‐Forskning och utbildning‐239 (A.A.); Clas Grochinskys minnesfond M2407, MF2513 (A.C.); Magnus Bergvalls Stiftelse 2024‐1063 (A.C.); Assar Gabrielssons Fond BGR23‐03 (A.C.); The Royal Society of Arts and Sciences in Gothenburg (A.A.), (A.C.); Axel Lennart Larssons donation (T.S.), Ivan o Eleonore Ljunggrens fond (T.S.), Wenner‐Gren Stiftelserna RSv2025‐0094 (T.S.); SWIMM, SSI, EFIS‐EJI (TS).

## Conflicts of Interest

The authors declare no conflicts of interest.

## Supporting information




**Supporting File**: eji70200‐sup‐0001‐SuppMat.pdf.

## Data Availability

The datasets analysed in this study are available in the NCBI Sequence Read Archive (SRA) under BioProject PRJNA277306 (accession code SRP055855). Any additional data supporting the findings of this study can be obtained from the corresponding author upon reasonable request.
