# Supplementary material for: Unfavourable H‐CDR3 Loops in preB Cells Lead to Highly Expanded Plasma Cell Clones
Source: Eur J Immunol. 2026 May 7;56:e70200. doi: 10.1002/eji.70200 (PMC13150949; doi:10.1002/eji.70200)
Supplement: Supplementary file 1 — Supporting File: eji70200‐sup‐0001‐SuppMat.pdf. [file EJI-56-e70200-s001.pdf]

Table S1. The primers used to sequence the BM B-cell and spleen/BM PC populations. The same forward primer was used for both BM B-cell populations and spleen/BM PCs, whereas different reverse primers were used, as indicated in the table.

	Forward	Sequence (5' to 3')	References
**BM B-cells & Spleen/BM PCs**	5' MsVHE	GGGAATTCGAGGTGCAGCTGCAGGAGTCTGG	1, 2
	**Reverse**	**Sequence (5' to 3')**	**References**
**BM B-cells**	Cm/inner or 3' Cμ inner	AGGGGGAAGACATTTGGGAAGGAC	1, 2
**Spleen/BM PCs**	IgG1/inner or 3' Cγ1 inner	GCTCAGGGAAATAGCCCTTGAC	1, 2
	IgG2b/inner or 3' Cγ2b inner	ACTCAGGGAAGTAGCCCTTGAC	1, 2
	3' Cγ2c inner	GCTCAGGGAAATAACCCTTGAC	2
	IgG3/inner or 3' Cγ3 inner	GCTCAGGGAAGTAGCCTTTGAC	1, 2

1.- Kantor et al., 1997, Handbook of Experimental Immunology, Blackwell Scientific Publications, pp. 13.1–13.6

2.- Tiller T., et al. J Immunol Methods*, 2009*

Table S2. Number of sequences of shared HE-PC clones. Amino acid sequences of highly expanded (HE) PC H-CDR3 clones from SLC$^{-/-}$ mice.

Organ and H-CDR3	n	V_H	Figure
**SPL**			
CTT IRY W	71	14-1	3A,B 4B
CTT IRY W	198	14-4	3A-C 4B
CTT LADY W	290	14-1	3A-C 4B
CTT LADY W	39	14-4	3A,B 4B
CAR RLLWFAY W	88	5-17	3A,B 4B
CAR RWLHYAMDY W	63	1-9	3A,B 4B
CAR RWLHYGMDY W	38	1-9	3A,B 4B
CAR RWGITTVVADWYFDV W	33	1-9	3A,B 4B
**BM**			
CTT IRY W	38	14-2	3A,B 4B
CTT IRY W	407	14-4	3A-C 4B
CST IRY W	389	14-4	3A-C 4B
CTT LADY W	42	14-1	3A,B 4B
CAR LGLLRLFDY W	42	14-1	3A,B 4B
CAR RWLHYAMDY W	93	1-9	3A,B 4B
CAR RWLHYGMDY W	43	1-9	3A,B 4B
CIR LYYDSPWFAS W	290	5-17	3A,B 4B
CIR LYYDSPWFAY W	55	5-17	3A,B 4B
CIR LYYDSPWFAT W	36	5-17	3A,B 4B
CAR DDGYLALMDY W	40	14-2	3A,B 4B
CAR GLLGSPYYFDY W	117	5-17	3A,B 4B

SPL (spleen); BM (bone marrow); PC (plasma cell); SLC$^{-/-}$ (surrogate light chain deficient); n (number of sequences).

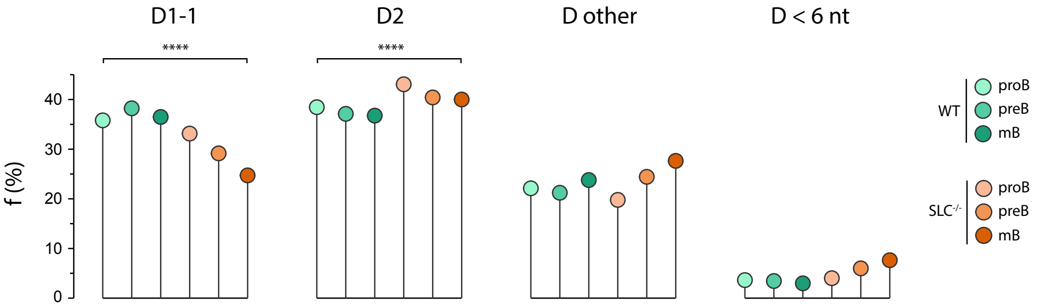

Figure S1. Lollipop plot showing the frequency of the D_H gene segments in BM B cells from WT and $SLC^{-/-}$ mice. Genotype comparison was carried out by collapsing D_H usage into two categories (D1-1, D2). Fisher's exact test was used for the analysis and p-values were corrected for multiple testing using the Holm method.
